# ISO 15189: 2012 : Quels changements pour les laboratoires africains?

**DOI:** 10.4102/ajlm.v4i1.181

**Published:** 2015-05-13

**Authors:** Nicolas Bouchet

**Affiliations:** 1 Quality Assurance Department, CNRFP (Centre National de Recherche et de Formation sur le Paludisme), Burkina Faso

## Introduction

La norme ISO 15189 est le standard international de référence en matière de biologie médicale,^1^ et l'accréditation est la reconnaissance d'un système qualité en pleine conformité avec cette norme.

Conscients des challenges actuels dans le domaine de la santé publique et de la recherche biomédicale, les laboratoires africains de biologie médicale pourraient être amenés à développer des systèmes de management de la qualité qui suivent les exigences de la norme ISO 15189. Sous l'impulsion de l'OMS, les programmes SLIPTA et SLMTA ont vu le jour ces dernières années en Afrique, pour accompagner les laboratoires dans la mise en place de processus qualité. ^2, 3, 4, 5^

Comme tout système qualité, les normes ISO évoluent, et sont périodiquement révisées; c'est en décembre 2012 que l'ISO, l'Organisation Internationale de Normalisation, a publié la troisième édition de la norme ISO 15189 ^6^, qui remplace la version de 2007. ^7^

Cet article a pour but de mettre en avant les changements majeurs entre les deux versions de cette norme, et les modifications que cela entraînera dans les systèmes qualité mis en place dans les laboratoires, et notamment les laboratoires africains.

### La norme ISO 15189: 2012

La norme dans sa version 2012 est un document plus clair et mieux organisé que la version 2007. Outre le titre qui s'est raccourci, la manière d'organiser les différentes rubriques a été changée, en suivant un ordre plus logique, ce qui permettra sans aucun doute aux utilisateurs de mieux appréhender les différentes exigences.

Lors de la présentation de la nouvelle version, l'ISO a parlé de « révision technique »^8^, cela signifiant un document présentant chaque aspect avec plus de détails; mais la lecture de cette nouvelle version montre que les changements sont en réalité plus conséquents.

### L'approche processus

Un processus est « un ensemble d'activités corrélées ou interactives qui transforme des éléments d'entrée en éléments de sortie »^9^. L'approche processus décrit l'organisation du travail comme une suite d’é tapes inter-reliées avec pour objectif la satisfaction optimale des clients et l'atteinte des objectifs fixés. Le but est de rendre l'organisation plus fonctionnelle, et moins dépendante de la hiérarchie, avec l'introduction et le développement du management transversal, de la culture du résultat et du travail d’équipe au sein du laboratoire.

La version 2007 abordait ce concept, mais laissait libre choix aux laboratoires de suivre ou non une approche processus dans leur système de management de la qualité (SMQ); cette approche existait déjà dans les SMQ basés sur la norme générale de la qualité, l'ISO 9001, qui avait introduit ce concept dans sa version 2000^1^, et l'avait maintenu dans sa version de 2008.^1^ La version 2012 de l'ISO 15189 est beaucoup plus explicite, l'approche processus devenant une exigence (sous-chapitre 4.2.1); ceci pourrait s'avérer compliqué à mettre en œuvre pour les laboratoires africains.

En effet, cette approche peut s'avérer difficile à mettre en place pour tous les laboratoires d'analyses de petite taille, avec un personnel peu nombreux, ces laboratoires représentant la majorité des laboratoires de biologie médicale en Afrique sub-saharienne. L'approche processus implique en effet de cartographier les processus, c'est-à-dire de représenter l'ensemble des processus nécessaires pour le SMQ et leurs séquences en déterminant les interactions qui les lient, et en désignant des pilotes de processus avec une définition très claire des rôles de chacun. Dans des structures de petite taille, une même personne peut avoir une vue d'ensemble et une vue détaillée des activités, et l'intérêt d'une cartographie précise des différents processus est souvent vu comme inutile. Si par exemple, dans un laboratoire de 4 employés, chaque individu est impliqué dans tous les processus, la mise en place d'une cartographie peut paraître superflue.

Néanmoins, cette approche présente plusieurs avantages: tout d'abord, elle insiste sur le but final du SMQ du laboratoire, le résultat du patient, pour permettre par la suite de bien définir le rôle de chacun dans l'obtention du résultat du patient, et de le montrer clairement. Elle permet enfin d'analyser et donc d'améliorer les performances du laboratoire.

Les laboratoires de biologie médicale ont un avantage pour mettre cette approche en œuvre, puisque leurs activités s'articulent autour de trois grands processus opérationnels, définis dans la norme: les processus pré-analytiques (chapitre 5.4), analytiques (5.5) et post-analytiques (5.7). Nous pensons qu'en se concentrant sur ces trois processus majeurs, un laboratoire pourra être en mesure de rattacher les autres processus (comme les équipements et la gestion des stocks, les ressources humaines, la satisfaction des clients, l'amélioration continue, etc.) à une cartographie assez simple.

### Manuel Qualité

Alors que pour la rédaction du manuel qualité, la version 2007 (sous-chapitre 4.2.4) proposait un plan en 23 points, la version 2012 s'attache à décrire (sous-chapitre 4.2.2.2) les six grands points essentiels dans un manuel qualité de laboratoire: politique qualité, description du SMQ, organisation et structure du laboratoire, rôles et responsabilités de la direction du laboratoire, système documentaire et les politiques établies pour le SMQ avec référence aux activités sur lesquelles elles reposent.

Ce changement va donc laisser à chaque laboratoire le soin de définir le plan de son manuel en fonction de son propre système qualité, ce qui démontrera d'une part la maîtrise de la norme par le laboratoire, et d'autre part la capacité du laboratoire à adapter la norme aux réalités locales, ce qui s'avère être souvent le cas en Afrique sub-saharienne. Le tout en simplifiant l’écriture de ce document, pierre angulaire du SMQ.

### Gestion des risques

Cet aspect est à nos yeux le changement majeur de la nouvelle version de la norme. Il apparaît dans le sous-chapitre 4.1.1.4, relatif au directeur de laboratoire, où il est du rôle du directeur d’ « élaborer et appliquer un plan de fonctionnement dégradé » ; il est précisé que ces plans doivent être soumis à essai de manière périodique. Le sous-chapitre 4.14.6 (Gestion des risques) aborde également le sujet, en mettant l'accent sur les risques de défaillances éventuelles sur les résultats des analyses; tout doit être mis en œuvre pour réduire et/ou éliminer ces risques. Le concept est également retrouvé dans le nouveau paragraphe consacré à la gestion des informations de laboratoire (5.10.3); il est précisé dans le point 5.3 que « le laboratoire doit disposer de plans de contingence […] en cas de défaillances ».

Ce concept de management des risques est une introduction importante de cette nouvelle norme; il implique un travail conséquent pour sa mise en œuvre, car toutes les situations d'urgence doivent être identifiées et évaluées (exemple: rupture en réactifs causée par un retard de livraison, panne d'un analyseur, coupure d’électricité, dysfonctionnement d'une imprimante, etc.). Chaque situation doit être inscrite dans ce « plan de fonctionnement dégradé », avec pour chacune:

La manière de résoudre ce problème (corrections = mesures
palliatives);
La manière de prévenir le patient et le
médecin;
La manière de communiquer le problème en interne;
Les actions correctives à mettre en œuvre pour éviter que
le problème ne se reproduise;
La manière de revenir à une situation normale.

Ces plans doivent ensuite être « testés périodiquement », et tout ce processus doit être documenté pour être justifié le cas échéant; ces tests doivent servir à améliorer le plan si nécessaire. Ces plans doivent également être audités en cas d'audit interne du système qualité.

Si l'on prend des exemples de risques comme une coupure d’électricité ou le retard d'approvisionnement en réactifs, ce sont des évènements rares dans les pays industrialisés, mais ce sont des problèmes courants dans les pays d'Afrique sub-saharienne. Nombreuses sont les capitales africaines qui vivent encore au rythme des délestages durant certaines périodes de l'année, et les retards de livraison sont fréquents pour tout ce qui concerne les réactifs et consommables dans les laboratoires. Ce qui sera un risque avec une probabilité d'apparition très faible dans un pays industrialisé sera un risque avec une fréquence d'apparition très élevée dans un pays de l'Afrique sub-saharienne, ce qui va rendre la gestion des risques selon la norme ISO plus difficile à mettre en œuvre pour les laboratoires africains. Les plans de fonctionnement dégradé devront être mis en œuvre périodiquement pour certains aspects, cela faisant qu'une opération prévue pour être « exceptionnelle » deviendra une opération de routine.

Il est à noter que cette introduction de la notion de gestion des risques dans la nouvelle version de la norme ISO 15189 s'inscrit dans une logique plus globale. Ce concept, qui vient du monde industriel, a fait ces dernières années son entrée dans le monde réglementaire.

Dans le cadre du processus de révision de la norme ISO 9001, qui est le référentiel de base en ce qui concerne les systèmes de management de la qualité (la nouvelle version est prévue pour 2015), le concept d'une approche de gestion des risques a été identifié par le groupe d'experts chargés de la révision de l'ISO 9001 (26ème réunion de l'ISO TC 176, Tokyo, février 2010)^8^, et sera intégré à la nouvelle version. La maîtrise des risques est clairement l'un des objectifs de cette nouvelle version de la norme ISO 9001, nécessitant de se projeter vers le futur pour anticiper tous les problèmes possibles qui pourraient empêcher la satisfaction client, objectif prioritaire de l'ISO 9001.

Dans le domaine même d'application de l'ISO 15189, dans les laboratoires de biologie médicale, ce concept a été introduit aux Etats-Unis par les nouvelles réglementations en matière de plan de contrôles de qualité internes, en suivant la ligne directrice du CLSI (*Clinical and Laboratory Standards Institute*) EP23-A.^12^ Cette ligne directrice a introduit le concept de gestion du risque dans le domaine des contrôles de qualité internes dans les laboratoires de biologie médicale^13, 14, 15^.

### Gestion des informations de laboratoire

La section 5.10 est une demi-nouveauté de cette version; il s'agit en effet de l'annexe B de la norme version 2007, qui passe donc du statut de ‘section informative’ à celui de ‘section normative’, avec obligation de mettre en œuvre ces exigences. Toute cette section est centrée sur la protection des données et la gestion du système d'information; le laboratoire doit s'assurer que les outils informatiques impliqués dans la gestion des informations de laboratoire (collecte, traitement, enregistrement, compte-rendu, conservation des données) soient validés par les fournisseurs, soient régulièrement maintenus et soient sécurisés. Les laboratoires devront tenir compte de ces nouvelles exigences et agir en conséquence, même si elles sont difficiles à mettre en œuvre, et tout aussi difficiles à documenter, encore plus dans notre contexte africain.

### Changements mineurs

Le point 4.14, qui était dans l'ancienne version de la norme uniquement consacré aux audits internes, est dans cette version 2012 plus complet, puisqu'il y est question de tous les aspects des audits et évaluations: revue des prescriptions, procédures et exigences concernant les échantillons, les évaluations des retours d'informations de la part des clients, la prise en compte des suggestions du personnel, les audits internes et externes, la gestion des risques et le suivi des indicateurs qualité.

Tous ce processus d’évaluation / audit a pour objectif d'améliorer le système qualité du laboratoire, en s'assurant que tous les processus nécessaires à la qualité des résultats des analyses ont été réalisés pour répondre aux exigences des clients.

Un nouveau sous-paragraphe (4.1.1.3) présente des règles d’éthique au sujet des conflits d'intérêts potentiels, de l'intégrité du personnel, des considérations éthiques dans le cadre de la manipulation des échantillons d'origine humaine et de la confidentialité des patients. Ce point est très important, car la culture de l’éthique des affaires / des entreprises est un concept peu développé en Afrique sub-saharienne, contrairement au concept d’éthique médical, qui était traité dans la version 2007 de la norme en Annexe C, ce qui a disparu dans la version 2012.

## Conclusion

La norme ISO 15189: 2012 reste le ‘*gold standard*’ en matière de qualité dans les laboratoires de biologie clinique. L'organisation plus claire de cette version permettra aux laboratoires de mieux s'imprégner du document pour mieux répondre aux exigences normatives. Il est en effet impératif aux laboratoires d'appliquer ce modèle qualité pour obtenir l'accréditation pour s'assurer la confiance des patients et un respect national et international.

Le défi est de taille pour les laboratoires africains, qui doivent s'adapter à cette norme en prenant en compte les conditions particulières dans lesquelles ils évoluent. Il est souhaitable que l'OMS-Afrique adapte sa liste SLIPTA à cette nouvelle version de la norme, en adaptant de manière générale tous les programmes d'accompagnement pour inclure tous les changements de la version 2012.
